# A New ELISA Using the ANANAS Technology Showing High Sensitivity to diagnose the Bovine Rhinotracheitis from Individual Sera to Pooled Milk

**DOI:** 10.1371/journal.pone.0145912

**Published:** 2016-01-13

**Authors:** Elisabetta Casarin, Laura Lucchese, Santina Grazioli, Sonia Facchin, Nicola Realdon, Emiliana Brocchi, Margherita Morpurgo, Stefano Nardelli

**Affiliations:** 1 University of Padova, Department of Pharmaceutical and Pharmacological Sciences Padova, Italy; 2 Istituto Zooprofilattico Sperimentale delle Venezie (IZSVe), Legnaro (PD), Italy; 3 Istituto Zooprofilattico Sperimentale della Lombardia ed Emilia Romagna (IZSLER), Brescia, Italy; 4 ANANAS nanotech, S.r.l., Padova, Italy; Universiti Malaysia Perlis, MALAYSIA

## Abstract

Diagnostic tests for veterinary surveillance programs should be efficient, easy to use and, possibly, economical. In this context, classic Enzyme linked ImmunoSorbent Assay (ELISA) remains the most common analytical platform employed for serological analyses. The analysis of pooled samples instead of individual ones is a common procedure that permits to certify, with one single test, entire herds as “disease-free”. However, diagnostic tests for pooled samples need to be particularly sensitive, especially when the levels of disease markers are low, as in the case of anti-BoHV1 antibodies in milk as markers of Infectious Bovine Rhinotracheitis (IBR) disease. The avidin-nucleic-acid-nanoassembly (ANANAS) is a novel kind of signal amplification platform for immunodiagnostics based on colloidal poly-avidin nanoparticles that, using model analytes, was shown to strongly increase ELISA test performance as compared to monomeric avidin. Here, for the first time, we applied the ANANAS reagent integration in a real diagnostic context. The monoclonal 1G10 anti-bovine IgG1 antibody was biotinylated and integrated with the ANANAS reagents for indirect IBR diagnosis from pooled milk mimicking tank samples from herds with IBR prevalence between 1 to 8%. The sensitivity and specificity of the ANANAS integrated method was compared to that of a classic test based on the same 1G10 antibody directly linked to horseradish peroxidase, and a commercial IDEXX kit recently introduced in the market. ANANAS integration increased by 5-fold the sensitivity of the 1G10 mAb-based conventional ELISA without loosing specificity. When compared to the commercial kit, the 1G10-ANANAS integrated method was capable to detect the presence of anti-BHV1 antibodies from bulk milk of gE antibody positive animals with 2-fold higher sensitivity and similar specificity. The results demonstrate the potentials of this new amplification technology, which permits improving current classic ELISA sensitivity limits without the need for new hardware investments.

## Introduction

In the context of animal health control and disease prevention it is fundamental to have access to efficient diagnostic tests capable of detecting early disease outbreaks and/or guaranteeing the absence of disease within large territorial extensions. Due to the large number of analyses that need to be routinely done within surveillance programs, such tests should be efficient, easy to use and, possibly, economical. Within serology analyses, high versatility and relatively low cost still make traditional microplate-based Enzyme Linked Immuno Sorbent Assay (ELISA) remaining the platform most widely used. However, in some cases, too low analyte levels make classic ELISA tests fail to meet the necessary performance needs, and higher sensitivity methods are necessary. For example, low antibody titers are common in some relevant veterinary diseases (e.g. during the prolonged phase of seroconversion to bovine paratuberculosis), or in pooled sample (sera or milk). With respect to this, bovine bulk milk is a typical example of a pooled sample widely used in veterinary diagnostics. Its collection is very cheap and easy, helping to reduce surveillance program costs by permitting to certify, with one single test only, entire herds as “disease-free”.

Limitations in the use of tank milk in comparison with the individual analyses on blood serum are linked to: a) the lower titres of antibodies physiologically present in the individual milk compared to the blood serum of the same animal; b) the dilution factor due to sample pooling in the bulk milk; c) fluctuations of the milk production during the lactation, which can decrease/increase the milk dilution ratio. Still, many ELISA tests developed for serum analysis can be extended to individual milk samples without significant loss in specificity or sensitivity. However, at herd level, extension to bulk milk is sometimes difficult to achieve [1–4]. As a general rule, the test must be sensitive enough to detect a single positive reaction in the pool. Currently, in the European Union—based on the requirements of the directive no. 1964/432/EEC- commercial ELISA reactions for Brucella and Enzootic Bovine Leukosis antibodies have been licensed by the National Reference Laboratories for the analysis of bulk milk of 100–200 cows. In the case of IBR, according to decision n. 2004/558/EC, to date the largest bulk pool size of the licensed kits corresponds to 50 cows.

The possibility to extend current bulk size limitations will depend only on the availability of more sensitive analytical tools. Indeed, in recent years, several new technological platforms for immunodiagnostic tests have been introduced, in general providing faster or more sensitive solutions than classic ELISA. However, most of these platforms require relatively large investments in instrumentation and/or sophisticated consumables, with an increase in costs that is not always sustainable in the veterinary and food control system.

**Fig 1:**
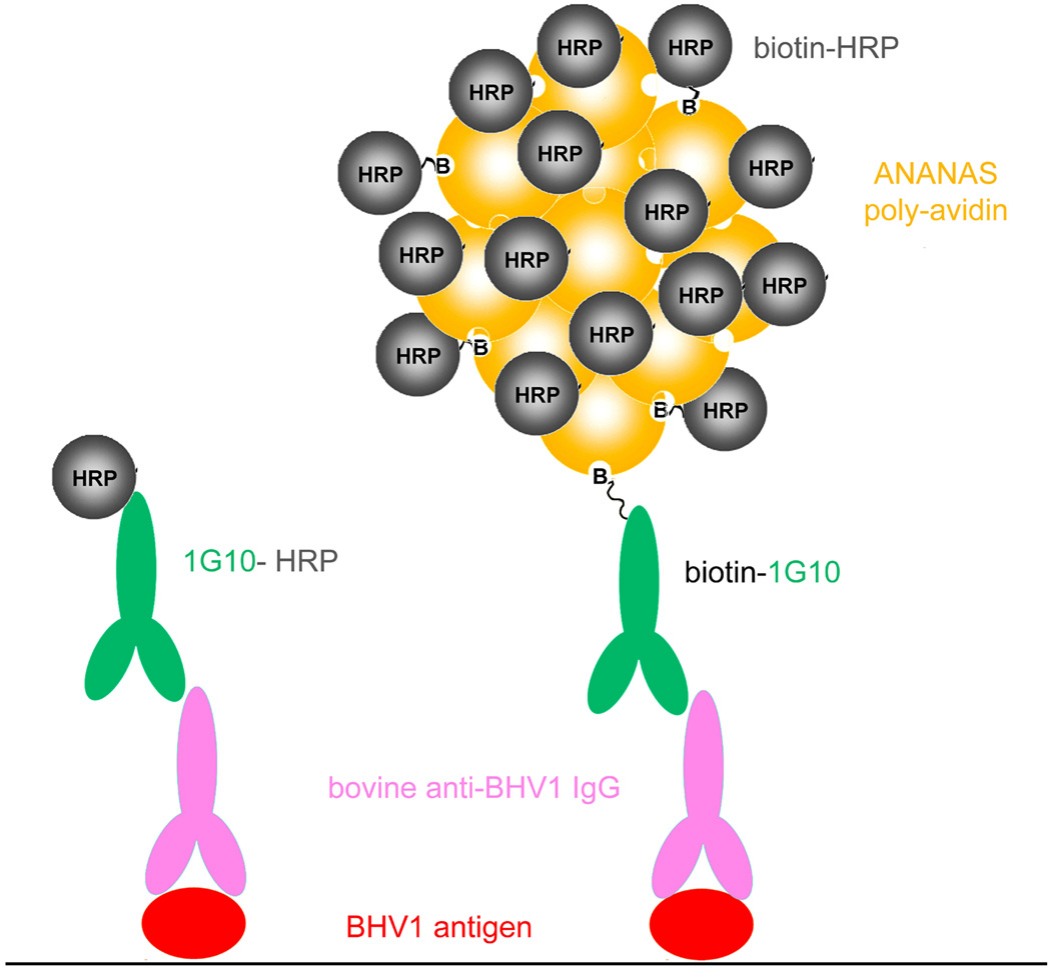
Cartoon (not in scale) depicting the two 1G10 mAb-based ELISAs for detection of anti BoHV-1 antibodies: classic configuration using the antibody-HRP conjugate (left) and ANANAS integrated method (right). For simplicity reasons, the mAb used for Bo-HV1 adsorption on the plate (see methods) is not included in the scheme.

An economically sustainable way to overcome sensitivity limitations in classic ELISA formats is to integrate signal amplifying reagents in the analytical process. For example, this is the case of the polymerized enzyme horseradish peroxidase (poly-HRPs) reagents, which in some cases are used as substitutes of the classic monomeric enzyme. A more recent ‘polymer’ based platform for signal enhancement is the one based on the Avidin-Nucleic-Acid-NanoASsemblies (ANANAS), a kind of poly-avidin nanoparticles, that can be used as alternative to monomeric avidin in several diagnostic applications [5–9]. The ANANAS nanoparticles (120 nm in diameter) are characterized by high stoichiometric control and contain about 350 functional assembled avidins. When used in combination with analyte-specific biotinylated antibodies and a biotinylated signal generating moiety, they bring to the immobilized analyte higher signal than monomeric avidin so that a strong signal amplification is generated (Fig 1). Compared to poly-HRPs, the ANANAS technology is more versatile as the reagents can be integrated with many signal generating technologies, including fluorescence, chemiluminescence or enzyme-generated colour [6,10,11].

In this work the ANANAS technology was integrated in an in-house trapping-indirect ELISA, originally developed for indirect diagnosis of Infectious Bovine Rhinotracheitis (IBR) through the detection of whole-virus antibodies in individual bovine blood sera (Fig 1). For this purpose, the anti-bovine IgG1 monoclonal antibody (1G10), classically employed as HRP conjugate in the conventional ELISA, was biotinylated and integrated with the ANANAS/biotin-HRP based detection reagents (Fig 1). Both detection methods were tested for their ability to diagnose IBR from milk samples. Sensitivity and specificity parameters were evaluated using reconstructed samples mimicking tank milk of different IBR prevalence (8-4-2 and 1%). For comparative purposes we included in our investigation a third kit, namely the commercial IBR Pool Ab Test from Idexx, recently introduced in the market, validated for both milk and serum testing.

IBR was selected for this study because a) it is a relevant disease, for which, currently, control programmes- based on bulk milk analysis- are ongoing in many countries; b) the reduced pool size (to date max 50 cows) of bulk milk samples which can be analyzed with the commercial ELISA kits currently available undermines the employment of the test in medium—large sized (>50 lactating cows) dairy farms.

IBR is an infectious disease caused by a bovine Herpesvirus (BoHV-1), which can be asymptomatic or cause mild to severe clinical signs [12–17]. The disease causes major economical losses in livestock production and reduces milk production, while the virus can be shed in semen and can be transmitted during natural and artificial insemination, propagating its effects onto next generations. For these reasons, control and eradication programs are actively being carried out in many regions in Europe [18,19], including North Eastern Italy, according to the legal provisions laid down in the 2004/558/EC decision.

While the reference BoHV-1 diagnostic method is the virus neutralization (VN) test [20], diagnosis through the detection of anti-BoHV-1 antibodies by ELISA (in serum or milk) [21,22] is less time consuming and more convenient to perform within large scale screenings [23]. Commercial competitive and non competitive ELISA kits validated for anti-BoHV-1 antibody detection in sera (individual or pool) or milk (individual or bulk) have been available for some years now. However, IBR bulk tests suffer from low sensitivity problems when using classic ELISA tools and amplification strategies are adopted, for example by concentrating the antibodies in the sample prior to analysis [24] (e.g. *cattletype* Milk Prep Kit by Qiagen and Eradikit^®^ IgG Milk Purification Kit by IN3 Diagnostic). In other cases, an amplification strategy has been inserted within the ELISA detecting reagents (IDEXX BHV-1 Bulk Milk Ab Test), leading to a high sensitive method. However, in this case, a “sensitive” washing step is required before signal development to prevent false positive readings. In addition, this test recommends the use of different cut-off values for data elaboration depending on the sample pool size, and this is difficult to perform in routine analyses as it requires tracking the size of each pool in the collection phase, dedicated software and/or qualified personnel for rough data elaboration. As of today, the issue of sensitivity in IBR diagnosis from bulk milk is not fully solved, justifying for the search for novel amplification solutions.

## Materials and Methods

### Reagents

#### BoHV-1 antigen

An historical respiratory strain of BoHV-1, isolated during ‘70s in Brescia province from a caw affected by IBR, was used as antigen in ELISA. Briefly, the virus was propagated in Aubek cell monolayers and harvested when cytopathic effect was maximum (24 h post-infection). The tissue culture harvest was centrifuged at 3000 *g* for 20 min and then inactivated with 0.001M Binary Ethyleneimine (BEI) for 48 h at 26°C; residual BEI was neutralized by sodium-thiosulphate at a final concentration of 2%.

The **anti-bovine IgG1** monoclonal antibody (mAb) 1G10 was developed at IZSLER. The 1G10-HRP conjugate was obtained through classic bioconjugation protocols. For integration in the ANANAS platform, mAb 1G10 was biotinylated through conventional conjugation protocols using a biotin *N*-hydroxysuccinimide derivative with a long (5KDa) poly(ethylene glycol) (PEG) spacer arm (Laysan Bio, AL, USA). ANANAS nanoparticles, biotin-HRP and ANANAS dilution buffer (DB) were kindly provided by ANANAS Nanotech (Padova, Italy, code# BioDe).

### Milk and blood sera samples

Milk (individual and bulk) and blood sera samples were already available as part of routine collection and analysis programs carried out for animal health surveillance and food testing activities at regional level. No specific permission was required for sample collection, since sampling actions took place for compulsory routinely controls (quality assessment of milk) or on the initiative of the trusted veterinarian (blood) for measuring the IBR immune coverage as well as virus circulation in vaccinated dairy herds. Blood samples were collected through caudal vein bleeding, according to the common veterinary practice. No animal was sacrificed.

**a. BoHV1 individual positive milk** samples were originating from cows (no. = 136) positive for IBR total antibodies in blood (ELISA test—SVANOVIR^®^ IBR Ab); according to the result for IBR-gE antibodies in blood (ELISA test—Idexx IBR gE Ab), positive cows were classified as either infected (no. = 66, IBR gE-antibody positive) or vaccinated (no. = 70, IBR gE-antibody negative); all infected cows were present in a farm when, 22 months before collecting the samples used for the present study, an IBR outbreak was diagnosed by means of PCR on nasal swabs; vaccinated cows were kept in a second farm, which was consistently negative for IBR gE antibodies over time. All cows—both infected and vaccinated—received a booster immunization with gE antibody-deleted marker vaccine every 6 months.

**b. Bulk BHV1- negative milk samples** were tank samples obtained from farms in the Bolzano province (Italy) which is free from IBR in accordance to Article 10 of Dir no. 1964/432/EEC, and whose cattle population is negative for IBR total antibodies. The average farm size in this province is small, with 5–10 lactating animals/herd.

**c. Negative diluting milk solution** was prepared by pooling negative tank samples of the Bolzano province.

After collection, all samples were added of sodium azide (NaN_3_) (final concentration 0.08 mg/mL). Individual negative and diluting milk solutions were stored at 4°C and used within 4 weeks from sampling. Positive samples were maintained at -20°C, thawed the day of testing, and serially diluted from 1:12.5 to 1:100 with negative diluting milk solution before analysis, thus simulating herd prevalence from 8% to 1%.

**Negative controls** were obtained from blood serum of a seronegative cow, diluted 1:50 (classic method with 1G10-HRP) or 1:5000 (ANANAS integrated method) in 1% yeast extract solution. **Positive controls** were dilutions of the serum from a seropositive animal, namely 1:50 or 1:800 in 1% yeast extract for the classic or the ANANAS-integrated methods respectively, pre-selected in order to yield, after 20 minutes of color development, 450 nm readouts of about 1.0 OD.

### ELISA procedures

**a.** Idexx IBR Pool Ab Test, Part Number: 99–55511 was used according to the manufacturer instructions.

b. Classic and ANANAS integrated methods

The test is a trapping-indirect ELISA in which the BoHV1 antigen is trapped onto the solid phase by an anti-BoHV1 mAb (mAb 4E5 developed at IZSLER). 96-well microplates (NUNC Maxisorp) were coated by overnight incubation at 4°C with an optimal concentration of the partially purified trapping mAb in carbonate/bicarbonate buffer, pH 9.6. After three washings with PBS containing 0.05% Tween 20 (PBST), the unpurified BoHV1 antigen was then dispensed in even columns (Ag+) at a predetermined saturating dilution in ELISA buffer (PBS pH 7.4, containing 1% yeast extract and 0.05% Tween 20—this buffer permits concurrent antigen immobilization and surface blocking). Odd columns received ELISA buffer alone(Ag^-^). The plates were then incubated for 1h at 37°C and washed again. These sensitized immunoplates were used immediately or were alternatively treated with a stabilizing solution, dried, sealed and stored at 4°C maintaining reactivity for over one year.

For the analysis, milk samples were incubated with the Ag^+^/Ag^-^ well pair, (45 min, 37°C) under gentle shaking, in parallel with positive and negative controls delivered in each plate. After washing with PBST, the presence of antibodies bound to the BoHV1 antigen was detected using: the anti-bovine IgG1 mAb 1G10, as HRP conjugate diluted in ELISA buffer (60 min, room temperature, RT) in the classic method; the same mAb 1G10 as biotin conjugate, diluted in DB (30 min, RT), followed (after washing with PBST) by the addition of ANANAS nanoparticles (60 min, RT) and (after washing again with PBST) biotin-HRP (30 min, RT) in the ANANAS integrated method. Nanoparticles and biotin-HRP (Fig 1) were used according to the manufacturer instructions.

After a final washings cycle with PBST, color development was obtained by distribution of a reday-to-use tetramethylbenzidine (TMB) solution (KPL, USA). Along the method optimization experiments, the reaction was blocked (0.5 M H_2_SO_4_) after 10 minutes. In all other experiments, color development was blocked when the signal at 620 nm of Ag+ well of the positive controls reached a value of about 0.4 OD (maximum 20 min) (corresponding to about 1.0 at 450 nm). Final plate reading was carried out at 450 nm. Standard volumes of 50 μL/well were used in each step of the reactions. Washing steps were carried out with Wellwash 4MK 2 microplate washer (ThermoFisherScientific, Waltham, MA, USA). Plate readouts were obtained with the Multiskan FC microplate reader (ThermoFisherScientific).

For result analysis, delta absorbance values (net OD) were calculated by subtracting the absorbance readings on Ag- wells from those of the corresponding Ag^+^ wells.

### Reagent optimization

To find out the best concentration of the biotinylated 1G10 antibody to be used in the ANANAS integrated assay, two positive samples of different titer were generated using the milk of a positive cow diluted to different extent (1:10 and 1: 100) in negative diluting milk. Data analysis was based on the analysis of the net OD and the absorbance registered at Ag- well (OD Ag-).

### Sensitivity Comparison Pilot study

Two small sets of positive samples from “vaccinated” (N = 8) or “infected” (N = 10) animals were analyzed upon dilution to different extent (1:25 and 1:50) in negative diluting milk solution and tested with both the ANANAS integrated and classic tests.

### Large Scale Validation

A large set of negative tank (N = 252) or positive individual (N = 136) samples—from vaccinated (N = 70) and infected (N = 66) animals—was used for the large validation experiment. Negative samples were tested as such, while positive samples were serially diluted in negative diluting milk solution (from 1:12.5 to 1:100) to mimic different values of seroprevalence in the group of lactating cows, (8, 4, 2, 1%) or different antibody levels, as expected in the field context. For data analysis, the absorbance readings were mathematically transformed into **S/P values** [1] according to: **S/P** = (net OD_sample_ / net OD_positive control_).

The non-parametric ROC curve was used to assess the diagnostic performance of the different serological kits and to define the cut-off values for data elaboration. The same set of samples was also analysed with a commercial ELISA kit (IBR Pool Ab Test) from Idexx Laboratories, Inc.; S/P values were calculated according to the manufacturer’s instructions [(OD_sample_—OD_neg control_) / (OD_pos control_−OD_neg control_)]; in addition to the manufacturer’s cut-off value (S/P > 0.25), results were evaluated using also the same cut-off value set for the home-made classic and ANANAS amplified ELISAs.

## Results and Discussion

### 1. ANANAS Integrated Reagent Optimization

**Fig 2:**
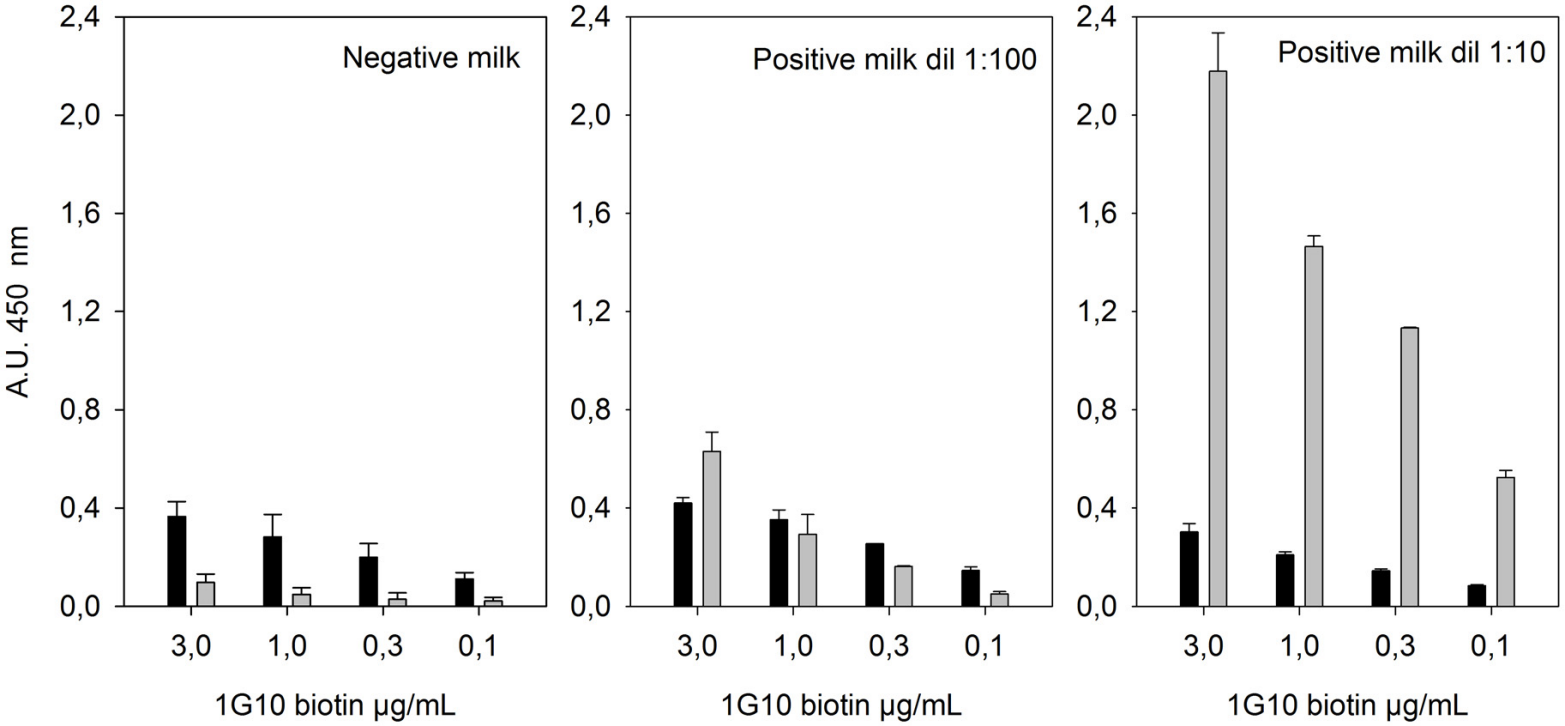
Optimization of detector mAb (1G10-biotin) concentration. **Net *OD*** (grey columns), **and OD*Ag***^***-***^ (black columns) registered in the analysis of negative diluting milk and an individual positive milk sample diluted 1:10 and 1:100 in negative diluting milk, as obtained with the ANANAS-integrated test using biotin-1G10 at different concentrations. TMB development was blocked after 10 min reaction. Each sample was analyzed in duplicate. These data are also reported in table format in (S1 Table).

The concentration of the detector biotinylated mAb 1G10 in the ANANAS-integrated test had to be optimized so to maximize positive signal and minimize noise-related background. Fig 2 displays the ***net OD*** and ***OD Ag***^***-***^ values obtained in the analysis of negative milk and a positive sample diluted in negative milk, as a function of biotinylated 1G10 concentration in the assay. The ***net OD*** value of the negative sample remained below 0.1 OD at all biotin-1G10 dilutions tested (between 0.1 and 3 μg/mL), while the ***net OD*** value of the positive samples was strongly correlated with the antibody concentration. This indicates that the ***net OD*** signal generated by the positive samples is specific. The plot of the ***net OD*** of the positive samples *vs* antibody concentration did not reach a *plateau*, indicating that the net signal could be further increased by using higher biotin antibody concentrations. However, also the signal generated on the Ag^-^ wells increased with the antibody concentration. The occurrence of this signal, which is probably related to non-specific interactions between 1G10 and some of the components used in plate conditioning, does not permit to increase indiscriminately the concentration of the antibody without generating a noisy plate. This non-specific signal—which could not be reduced even using extensive washing steps or by changing the plate blocking media- is disturbing, especially when testing low titer samples (e.g. the 1:100 dilution). Based on these experiments a concentration of 2 μg/mL was selected for all further experiments.

### 2. Sensitivity Comparison Pilot study

**Fig 3:**
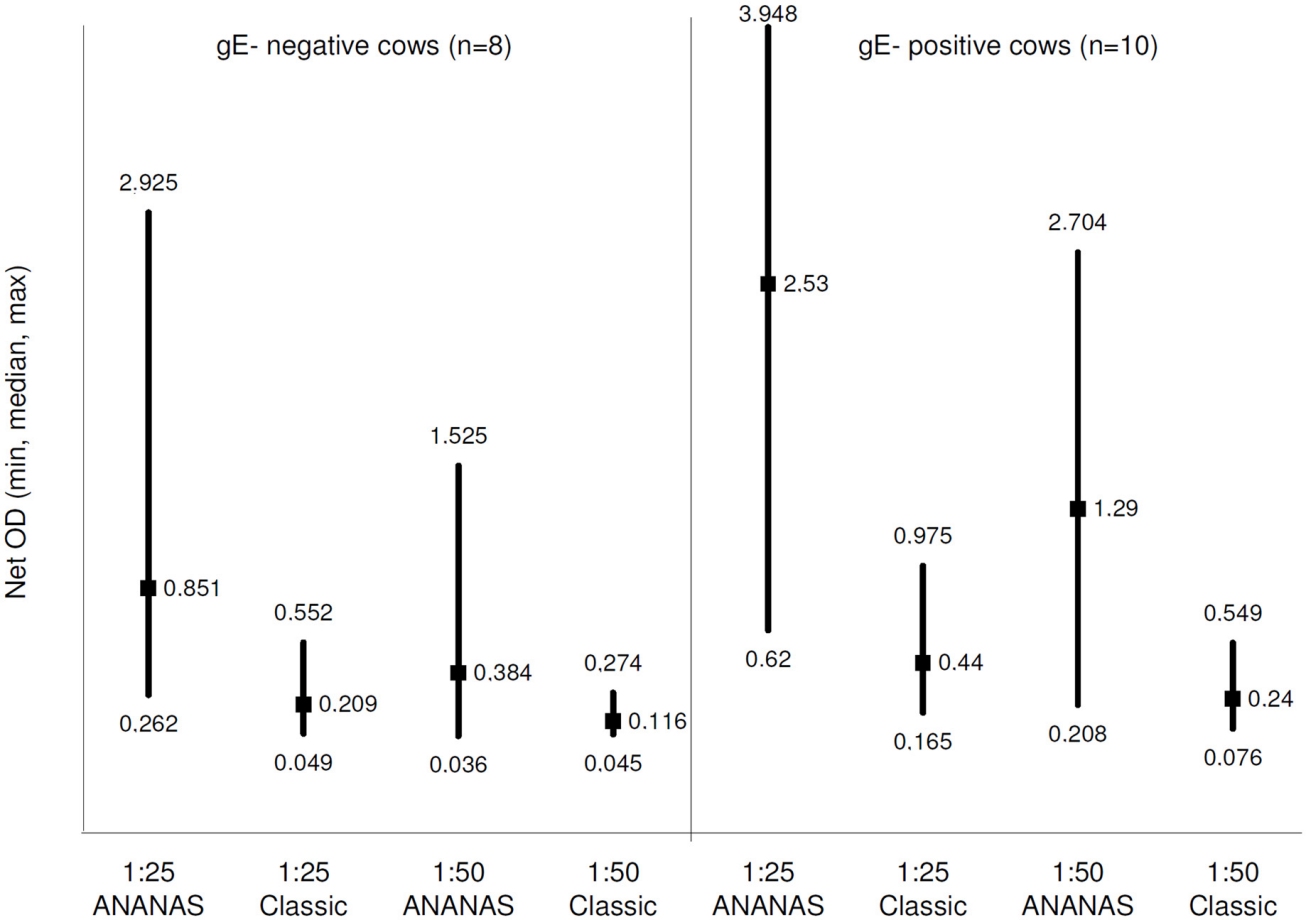
Assay sensitivity comparison in the pilot study. Maximum, minimum and median *net OD* values registered in the analysis of individual positive milk samples (no. 8 from gE-negative and no. 10 from gE-positive cows) diluted 1:25, 1:50 in negative diluting milk, using 1G10 in the classic or ANANAS-integrated configuration.

A second preliminary investigation was carried out to quantify the amplification potential of the ANANAS integration. As summarized in Fig 3, the ***net OD*** generated by using the ANANAS integrated method was about 5-fold higher than that generated by the classic one. Glycoprotein E-antibody positive animals showed a higher reactivity than gE antibody-negative ones, likely due to the different origin of their immune response (infection + vaccination *vs* vaccination only).

### 3. Large Scale Validation

Initially, for both classic and ANANAS integrated tests, we calculated the cut-off values which minimize the distance between the curve and upper left corner of the ROC curves, and the Diagnostic Sensitivity (DSe), the Diagnostic Specificity (DSp) and the Area Under Curve (AUC) were calculated using such values. Considering the whole sample population, this analysis gave (see also S2 Table), for the ANANAS integrated method: Cut-off S/P = 0.04; Dse = 0.86 –Dsp = 0.90; and for the classic method: Cut-off S/P = 0.02, Dse = 0.81 –Dsp = 0.85. Notably, these Dsp values are too low for speculating about a routinary use of the tests, because of the high frequency of false positive results. In fact, a bulk milk test for IBR antibodies is tipically employed for substantiating the freedom from the disease in a negative population and, for this reason, a 10–15% of false positive results is not acceptable. Therefore, we recalculated specifity and sensitivity values upon setting the cut-off value for positive samples at S/P > 0.1. This value was selected and used for all further data analyses because a) for both methods, it represents the mean S/P of negative samples + 3 standard deviations, and b) it is the lowest value associated with a practically significant net OD (about 0.15–0.20). Using this threshold, the observed specificity is > 0.995 (Table 1), which is a much more useful value for screening negative populations. By the way, due to the small size of the dairy farms of the Bolzano province, individual milk of false-positive reactors is less diluted, thus increasing the likelihood of false-positive bulk milk results. In many European areas geared to dairy production the average farm size is larger and, consequently, specificity values of the test should be higher.

**Table 1:** ELISA test specificity comparison. Assay specificity calculated for the classic method based on 1G10-HRP conjugate, the 1G10-ANANAS integrated, and the commercial IDEXX one. Results of the IDEXX test were interpreted adopting the S/P threshold indicated by the manufacturer (0.25) or the same one adopted for the 1G10 based methods (0.1). The results have been obtained using 252 negative tank milk samples from farms in the Bolzano province

TEST	Specificity
ANANAS (S/P threshold >0.10)	99.6%
Classic (S/P threshold >0.10)	99.6%
Idexx (S/P threshold >0.25)	100%
Idexx (S/P threshold >0.10)	100%

Noteworthy, specificity, which depends mostly on the quality of the antibody-antigen recognition event, was comparable for the two 1G10 mAb-based ones. The fact that the specificity of 1G10-based classic test was maintained upon ANANAS integration indicates that the long PEG spacer biotinylation necessary for optimal ANANAS nanoparticle integration does not alter 1G10 antigen recognition.

**Fig 4:**
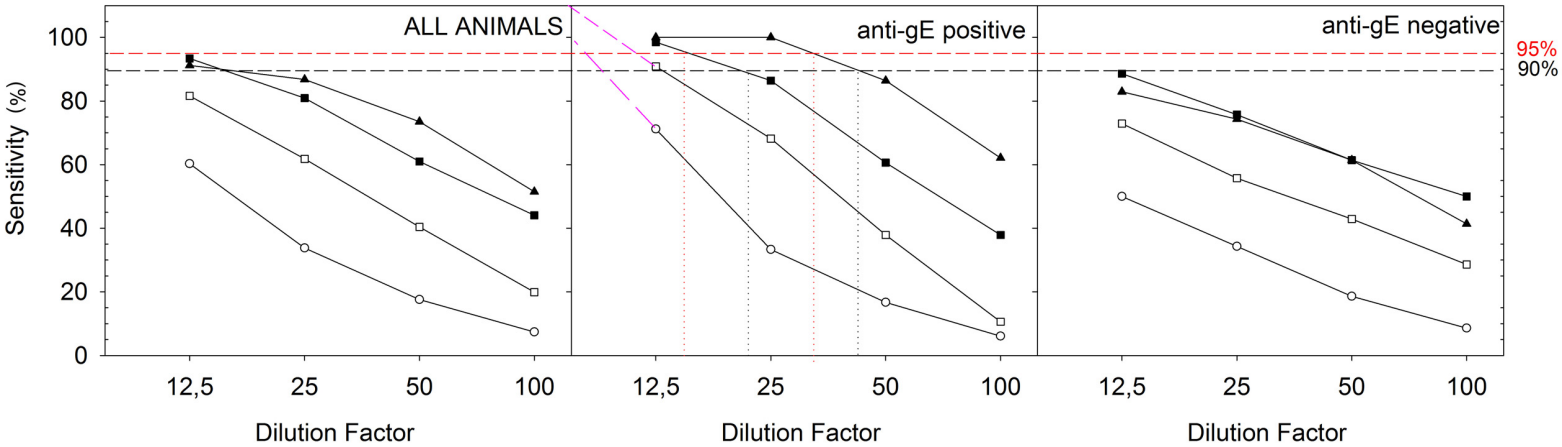
ELISA tests sensitivity comparison. Sensitivity as a function of milk dilution using the (o) classic test, (▲) ANANAS integrated, (□) Commercial with 0.25 S/P threshold, (■) commercial with 0.1 S/P threshold methods. Data have been elaborated taking into consideration all animals (left panel) or separating the results of anti-gE positive (mid panel)/negative (right panel) animals. The dashed parts of the curves in mid panel were generated by linear extrapolations from the two closest data points. The vertical dotted lines highlight the interception of dilution curves with the 95/90% sensitivity. These data are also reported in table format in (S3 Table).

As to the sensitivity, data were analyzed both collectively and considering separately sample dilution and gE-antibody positivity/negativity status. Fig 4 displays the relationship between sample dilution and sensitivity for the three tests, taking into account collected samples or gE-antibody positive/negative samples separately.

In general, ANANAS integration increased dramatically 1G10 mAb-based test sensitivity. The difference between classic and ANANAS integrated tests is such that no statistical support was necessary to demonstrate significance. The ANANAS-integrated method was also significantly more sensitive than the commercial kit using the S/P threshold indicated by the manufacturer (0.25). When adopting the same S/P threshold (0.1) for the two tests, similar sensitivity was registered when taking into consideration the full sample population.

Interestingly, when analyzing separately the results on gE-antibody positive/negative animal samples, the ANANAS integrated method was consistently more sensitive on samples from gE-antibody positive animals regardless of the dilution factor (see also Supplementary Information, S3 Table), in line with what observed along the sensitivity comparison pilot study (Fig 3). On the other hand, the commercial kit showed higher sensitivity for gE-antibody positive samples at the 1:12.5 and 1:25 dilutions, but at higher dilutions (1:50 and 1:100) the sensitivity parameter was either independent of the gE-antibody positivity/negativity status or even more favourable towards the gE-antibody negative samples. Likely, the difference between the two tests is unrelated to the signal amplification strategy, and we can speculate it may be related to differences in antigen presentation and/or reactivity of the anti-bovine immunoglobulin detector. In the plates used with the classic and ANANAS integrated 1G10 mAb based methods, whole inactivated BoHV-1 were immobilized so to be able to capture all of the IBR related immunoglobulins present in the sample. The BoHV-1 antigen used in the commercial kit is not indicated by the manufacturer, so we can only speculate that it is capable of collecting a smaller subset of IBR related antibodies. It is important to note that along surveillance programs it is more important to detect gE-antibody positive (infected) animals than gE-antibody negative (vaccinated) ones: indeed vaccinated animals testing false negative do not exert any consequence in terms of epidemiological risk.

Extrapolation of anti-gE-antibody positive sample data indicates that the dilution limits yielding 95%/90% sensitivity are 1:34/1:44, 1:16/1:21 and 1:4/1:6 for the ANANAS-integrated, commercial IDEXX with 0.1 S/P threshold, and classic methods, respectively. In other word, the ANANAS-integrated 1G10 method is two-fold more sensitive than the commercial kit and between 6–11 fold more sensitive than the original in-house ELISA in identifying IBR related IgGs from milk of anti-gE-antibody positive (infected) animals.

## Conclusions

The results showed that it is feasible to improve a test developed for single serum testing to pooled milk, by switching from an antibody-HRP conjugate to the ANANAS integrated detection method. The system is convenient to use and does not require novel instrumentation investments. In this case, about 5-fold increase in analytical sensitivity was achieved by replacing the antibody conjugate with the ANANAS integrated detection. It is worth noticing that, in other contexts and using model analytes, ANANAS integration was capable of improving sensitivity more than the value obtained here. Indeed, the efficacy of an amplification system also depends on the quality of the primary recognition, as amplification involves both signal and noise. In this case, the ANANAS amplification potential had to be down regulated to avoid the increase of noise due to non-specific interaction of the detector anti-bovine IgG1 mAb 1G10 with the coated plate. Nevertheless, the method here developed was more sensitive than the commercial kit in detecting IBR related IgGs from animals that were subject to a disease outbreak during their lifetime, with the capability of detecting 2.5% prevalence with more than 90% sensitivity.

From a more general point of view, the results prove that the ANANAS platform can be used as a valid alternative to other amplification systems currently adopted to improve ELISA tests that suffer from sensitivity issues. Further advantage of the ANANAS reagents is that they can be integrated with signal generating systems others than the peroxidase, such as the alkalin phosphatase or natural or synthetic fluorophores. This permits to expand its amplification potential to analytical platforms different than ELISA. Investigation in this direction is part of ongoing investigations.

## Supporting Information

S1 Table Net OD, and ODAg-Neg registered in the analysis of negative diluting milk and of individual positive milk diluted in negative diluting milk, as obtained with the ANANAS-integrated test using biotin-1G10 at different concentration. (DOC)

S2 Table Cut-off value, Diagnostic Sensitivity (DSe), Diagnostic Specificity (DSp) and Area Under Curve (AUC) of the classic and the ANANAS-integrated Elisa methods calculated with non-parametric ROC curve analysis. (DOC)

S3 Table Sensitivity (and 95% confidence intervals) of the three ELISA tests calculated according to specified thresholds. (DOC)
