# Supplementary material for: A New ELISA Using the ANANAS Technology Showing High Sensitivity to diagnose the Bovine Rhinotracheitis from Individual Sera to Pooled Milk
Source: PLoS One. 2016 Jan 13;11(1):e0145912. doi: 10.1371/journal.pone.0145912 (PMC4712047; doi:10.1371/journal.pone.0145912)
Supplement: S1 Table — (DOC) [file pone.0145912.s001.doc]

**S1 Table.**

***Net OD*, and OD*Ag^-^Neg* registered in the analysis of negative diluting milk and of individual positive milk diluted in negative diluting milk, as obtained with the ANANAS-integrated test using biotin-1G10 at different concentration.**

|  | **Negative milk** | | **(milk diluted 1:10 in negative diluting milk)** | | **(milk diluted 1:100 in negative diluting milk)** | |
| --- | --- | --- | --- | --- | --- | --- |
| **1G10 biotin µg/mL** | **OD450 Ag^-^** | **Net OD** | **OD450 Ag^-^** | **Net OD** | **OD450 Ag^-^** | **Net OD** |
| 3 | **0.365+/-0.061** | 0.097+/-0.034 | 0.303+/-0.034 | 2.178+/-0.157 | 0.420+/-0.023 | 0.631+/-0.078 |
| 1 | **0.283+/-0.091** | 0.048+/-0.027 | 0.209+/-0.013 | 1.465+/-0.043 | 0.352+/- 0.040 | 0.293+/-0.081 |
| 0.3 | **0.198+/-0.057** | 0.028+/-0.026 | 0.144+/-0.008 | 1.133+/-0.003 | 0.255+/- 0.000 | 0.162+/-0.003 |
| 0.1 | **0.111+/-0.026** | 0.021+/-0.015 | 0.084+/-0.004 | 0.525+/-0.028 | 0.146+/-  0.015 | 0.050+/-0.010 |

(*) TMB development was stopped after 10 min reaction. Each sample was analyzed in duplicate.
