# Supplementary material for: A New ELISA Using the ANANAS Technology Showing High Sensitivity to diagnose the Bovine Rhinotracheitis from Individual Sera to Pooled Milk
Source: PLoS One. 2016 Jan 13;11(1):e0145912. doi: 10.1371/journal.pone.0145912 (PMC4712047; doi:10.1371/journal.pone.0145912)
Supplement: S2 Table — (DOC) [file pone.0145912.s002.doc]

**S2 Table.**

**Cut-off value, Diagnostic Sensitivity (DSe), Diagnostic Specificity (DSp) and Area Under Curve (AUC) of the classic and the ANANAS-integrated Elisa methods calculated with non-parametric ROC curve analysis.**

| **REFERENCE SAMPLE POPULATION** | | **ELISA test** | **Cut-off** | **DSe** | **DSp** | **AUC** |
| --- | --- | --- | --- | --- | --- | --- |
| Negative | Positive |  |  |  |  |  |
| ALL BULK SAMPLES  (n = 252) | ALL ANIMALS  (n = 544,  from 136 animals)* | ANANAS | 0.0449 | 0.8621 | 0.8968 | 0.9198 |
|  |  | CLASSIC | 0.0166 | 0.8107 | 0.8532 | 0.9043 |
| ALL BULK SAMPLES (n = 252) | gE antibody POS  (n = 264,  from 66 animals)* | ANANAS | 0.0711 | 0.9015 | 0.9524 | 0.9631 |
|  |  | CLASSIC | 0.0201 | 0.8636 | 0.9008 | 0.9424 |
| ALL BULK SAMPLES (n = 252) | gE antibody NEG  (n = 280,  from 70 animals)* | ANANAS | 0.0454 | 0.7964 | 0.8968 | 0.879 |
|  |  | CLASSIC | 0.0158 | 0.7536 | 0.8333 | 0.8684 |
| ALL BULK SAMPLES (n = 252) | DILUTION 1:12.5  (n = 136) | ANANAS | 0.0757 | 0.9265 | 0.9643 | 0.9637 |
|  |  | CLASSIC | 0.0268 | 0.9265 | 0.9365 | 0.9734 |
| ALL BULK SAMPLES (n = 252) | DILUTION 1:25  (n = 136) | ANANAS | 0.0624 | 0.9265 | 0.9365 | 0.9633 |
|  |  | CLASSIC | 0.0207 | 0.8750 | 0.9048 | 0.9292 |
| ALL BULK SAMPLES (n = 252) | DILUTION 1:50  (n = 136) | ANANAS | 0.0494 | 0.8456 | 0.9008 | 0.9161 |
|  |  | CLASSIC | 0.0166 | 0.8162 | 0.8532 | 0.894 |
| ALL BULK SAMPLES (n = 252) | DILUTION 1:100  (n = 136) | ANANAS | 0.0290 | 0.7721 | 0.8294 | 0.8359 |
|  |  | CLASSIC | 0.0119 | 0.7353 | 0.7500 | 0.8207 |
