# Supplementary material for: A New ELISA Using the ANANAS Technology Showing High Sensitivity to diagnose the Bovine Rhinotracheitis from Individual Sera to Pooled Milk
Source: PLoS One. 2016 Jan 13;11(1):e0145912. doi: 10.1371/journal.pone.0145912 (PMC4712047; doi:10.1371/journal.pone.0145912)
Supplement: S3 Table — (DOC) [file pone.0145912.s003.doc]

**S3 Table**

Sensitivity (and 95% confidence intervals) of the three ELISA tests calculated according to specified thresholds.

| **ELISA test** | **SAMPLES** | **1:12.5** | **1:25** | **1:50** | **1:100** |
| --- | --- | --- | --- | --- | --- |
| CLASSIC  (S/P Threshold 0.10) | ALL ANIMALS (n = 136) | 60.3  (68.5-51.5) | 33.8  (42.5-26.1) | 17.6  (25.3-11.8) | 7.4  (13.5-3.8) |
|  | gE antibody POS (n = 66) | 71.2  (81.4-58.6) | 33.3  (46.1-22.5) | 16.7  (28.3-9.0) | 6.1  (15.6-2.0) |
|  | gE antibody NEG (n = 70) | 50.0  (62.1-37.9) | 34.3  (46.7-23.6) | 18.6  (30.0-10.6) | 8.6  (18.4-3.5) |
| ANANAS  (S/P Threshold 0.10) | ALL ANIMALS (n = 136) | 91.2  (95.2-84.8) | 86.8  (91.8-79.6) | 73.5  (80.5-65.2) | 51.5  (60.1-42.8) |
|  | gE antibody POS (n = 66) | 100.0  (100.0-93.1) | 100.0  (100-93.1) | 86.4  (93.2-75.2) | 62.1  (73.5-49.3) |
|  | gE antibody NEG (n = 70) | 82.9  (90.5-71.6) | 74.3  (83.7-66.2) | 61.4  (72.6-49.0) | 41.4  (53.8-30.0) |
| IDEXX  (S/P Threshold –  according to manufacturer) | ALL ANIMALS (n = 136) | 81.6  (87.5-73.9) | 61.8  (69.8-53.0) | 40.4  (49.2-32.2) | 19.9  (27.7-13.7) |
|  | gE antibody POS (n = 66) | 90.9  (96.3-80.6) | 68.2  (78.8-55.4) | 37.9  (50.7-26.5) | 10.6  (21.2-4.7) |
|  | gE antibody NEG (n = 70) | 72.9  (82.5-60.7) | 55.7  (67.4-43.4) | 42.9  (55.2-31.3) | 28.6  (40.8-18.7) |
| IDEXX  (S/P Threshold 0.10) | ALL ANIMALS (n = 136) | 93.4  (96.7-87.4) | 80.9  (86.9-73.1) | 61.0  (69.2-51.3) | 44.1  (52.9-35.7) |
|  | gE antibody POS (n = 66) | 98.5  (99.9-90.7) | 86.4  (93.2-75.2) | 60.6  (72.2-47-8) | 37.9  (50.7-26.5) |
|  | gE antibody NEG (n = 70) | 88.6  (94.6-78.2) | 75.7  (84.8-63.7) | 61.4  (72.6-49.0) | 50.0  (62.1-37.9) |
